# Utilisation of Chimeric Lyssaviruses to Assess Vaccine Protection against Highly Divergent Lyssaviruses

**DOI:** 10.3390/v10030130

**Published:** 2018-03-15

**Authors:** Jennifer S. Evans, Guanghui Wu, David Selden, Hubert Buczkowski, Leigh Thorne, Anthony R. Fooks, Ashley C. Banyard

**Affiliations:** 1 Wildlife Zoonoses and Vector Bourne Disease Research Group, Animal and Plant Health Agency, Woodham Lane, Weybridge, Surrey KT15 3NB, UK; jenni_s_evans@hotmail.co.uk (J.S.E.); guanghui.wu@apha.gsi.gov.uk (G.W.); DAvid.selden@apha.gsi.gov.uk (D.S.); hubert.buczkowski@gazeta.pl (H.B.); leigh.thorne@apha.gsi.gov.uk (L.T.); tony.fooks@apha.gsi.gov.uk (A.R.F.); 2 University of Warwick, Gibbet Hill Road, Coventry, West Midlands CV4 7AL, UK; 3 Institute for Infection and Immunity, St. George’s Hospital Medical School, University of London, London SW17 0RE, UK

**Keywords:** lyssavirus, rabies, vaccine, neutralizing antibody, chimera, antigenic

## Abstract

Lyssaviruses constitute a diverse range of viruses with the ability to cause fatal encephalitis known as rabies. Existing human rabies vaccines and post exposure prophylaxes (PEP) are based on inactivated preparations of, and neutralising antibody preparations directed against, classical rabies viruses, respectively. Whilst these prophylaxes are highly efficient at neutralising and preventing a productive infection with rabies virus, their ability to neutralise other lyssaviruses is thought to be limited. The remaining 15 virus species within the lyssavirus genus have been divided into at least three phylogroups that generally predict vaccine protection. Existing rabies vaccines afford protection against phylogroup I viruses but offer little to no protection against phylogroup II and III viruses. As such, work involving sharps with phylogroup II and III must be considered of high risk as no PEP is thought to have any effect on the prevention of a productive infection with these lyssaviruses. Whilst rabies virus itself has been characterised in a number of different animal models, data on the remaining lyssaviruses are scarce. As the lyssavirus glycoprotein is considered to be the sole target of neutralising antibodies we generated a vaccine strain of rabies using reverse genetics expressing highly divergent glycoproteins of West Caucasian Bat lyssavirus and Ikoma lyssavirus. Using these recombinants, we propose that recombinant vaccine strain derived lyssaviruses containing heterologous glycoproteins may be a suitable surrogate for wildtype viruses when assessing vaccine protection for the lyssaviruses.

## 1. Introduction

The lyssavirus genus is a group of high consequence pathogens with, following the onset of clinical disease, a near 100% fatality rate. Rabies virus (RABV), the prototype lyssavirus, causes over 59,000 human deaths annually, with the majority of the fatalities being in Africa and Asia [1]. This figure is believed to be a gross underestimate as a large proportion of rabies deaths occur in resource limited areas that lack medical facilities and adequate reporting systems to record human cases of rabies [2,3]. Further, in endemic areas, diagnostic capabilities are also often lacking and as such syndromic assessment is used to determine rabies, often with patients showing symptoms of disease being sent home to die [3,4]. Despite the burden of this horrific disease, rabies continues to be considered a neglected tropical disease with little support available in the majority of endemic regions to try and reduce the burden of disease [5].

RABV is just one member of a divergent genus of viruses that are all thought capable of causing fatal encephalitis in any infected mammal [5]. The main mode of transmission is through mechanical transfer, most often following the bite of an infected dog. However, interestingly these pathogens have been isolated across the globe from a variety of mammalian species with 14 of the 16 proposed lyssavirus species being detected in bats [6]. Interestingly, only non-rabies lyssaviruses have been detected in bats across the Old World with bat rabies virus being apparently restricted to the New World [7]. Despite this, rabies in terrestrial mammals has been detected globally with very few isolations of lyssaviruses in terrestrial species and no evidence of sustained lyssavirus transmission in terrestrial carnivores [8].

Within this genus, the 16 recognized lyssavirus species are genetically and antigenically divided into phylogroups that generally dictate the ability of rabies vaccines to afford protection. Serological responses to RABV vaccination are measured by quantitating the neutralisation of virus using test sera through comparison with neutralisation by control sera. All human rabies vaccines are based on inactivated preparations of whole virus. The response to vaccination can differ between individuals but it is widely accepted that the protective cut off for serological positivity and protection against RABV is 0.5 international units (IU)/mL [9]. Phylogroup I includes the classical RABVs alongside Aravan lyssavirus (ARAV), Australian bat lyssavirus (ABLV), Bokeloh bat lyssavirus (BBLV), Duvenhage lyssavirus (DUVV), European bat-1 and -2 lyssaviruses (EBLV-1 and EBLV-2), Irkut lyssavirus (IRKV), Khujand lyssavirus (KHUV), and Gannoruwa bat lyssavirus (GBLV). Studies with some of these phylogroup I viruses have demonstrated that a serological titre of 0.5 IU/mL is not sufficient for protection but that higher titres can neutralise [10,11,12,13,14]. In contrast, the antibody response generated by vaccination is generally considered to be insufficient to confer protection against the more genetically and antigenically divergent lyssaviruses. Phylogroup II encompasses the African Lyssaviruses, and includes Lagos bat lyssavirus (LBV-lineages A-D), Mokola lyssavirus (MOKV), and Shimoni bat lyssavirus (SHIBV) whilst even more divergent viruses have been proposed as belonging to phylogroup III [15] and include West Caucasian bat lyssavirus (WCBV), Ikoma lyssavirus (IKOV) and Lledia bat lyssavirus (LLEBV) [16]. In vivo vaccination-challenge experiments have shown reduced or no efficacy of current licensed rabies vaccines against viruses in phylogroup II (MOKV, LBV, SHIBV) [12], phylogroup III (WCBV) [10,17] and the most divergent lyssavirus characterised to date, IKOV. Where available, interactions between viruses and sera have been quantitated using antigenic cartography and have reiterated the antigenic distances between the three phylogroups [18].

As the target for neutralising antibody responses against all lyssaviruses is the surface glycoprotein (G) [19], the glycoproteins from divergent lyssaviruses were swapped into a vaccine backbone to enable vaccination challenge experiments to be performed with reduced risk to the operator and without compromising experimental outputs assessing serological protection from vaccination. Here we describe the generation of recombinant rabies vaccine strain viruses containing divergent glycoproteins and demonstrate their utility in vaccination challenge experimentation with reduced risk to the operator. The exchange of glycoproteins was tolerated in vitro with peak titres being comparable to previous reports with wildtype viruses [17]. In vivo, a lack of protection from existing vaccines was demonstrated in mice that had been vaccinated with rabies vaccines and challenged with the chimeric viruses. Furthermore, peripheral inoculation with the chimeric viruses resulted in survivorship indicating that these recombinants may exhibit reduced pathogenicity when compared to wildtype viruses although further assessments of wildtype viruses is warranted. These chimeric viruses may be considered as an alternative option for studying RABV vaccine protection against highly divergent lyssaviruses in vivo as their pathogenicity may be reduced.

## 2. Materials and Methods

### 2.1. Full Length Plasmid Construction

The rabies vaccine strain (SN) reverse genetics DNA backbone was utilised for all recombinant virus genome clone assembly. The SN strain is based on the street Alabama Dufferin (SAD) B19 vaccine strain of rabies as described previously [20,21,22,23,24]. Constructs were generated by exchanging the vaccine strain G with that of the highly divergent West Caucasian Bat virus G and the Ikoma virus G (Figure S1). The parent homologous cSN strain was rescued and characterised alongside the recombinant viruses. Restriction endonuclease (REs) sites, *HpaI* and *NheI* (Promega, Madison, WI, USA) were utilised to facilitate exchange of the G open reading frames (ORFs). The recombinant G ORFs were amplified, using primers that incorporate the respective RE sites, from total cellular RNA from lyssavirus infected cells (WCBV G transfer into FL *HpaI* For: 5′ TATATATAGTTAACAAGATGGCTTCCTACTTTGC 3′; WCBV G transfer into FL *NheI* Rev: 5′ TATATATAGTCAGCACCTTGTTATTGGGCAGTTTGTC; IKOV G transfer into FL *HpaI* For: 5′ TATATATAGTTAACAAGATGGCTCAGTTGGTCAC 3′; IKOV G transfer into FL *NheI* Rev: 5′ TATATATAGTCAGCAACCCACTAGAATGCAGAACTCTTG 3′). Following digestion, gel purification, ligation, and transformation, clones were checked by restriction enzyme digestion and plasmid sequencing prior to virus rescue.

### 2.2. Virus Rescue, Titration, and Growth Curves

Virus rescue was performed within SAPO4/ACDP3 biocontainment facilities as described previously [21,25] using fowlpox T7 (FPT7) to provide sufficient T7 RNA polymerase. Baby Hamster Kidney cells (BHKs) were infected with FPT7 at a multiplicity of infection (moi) of 0.01 for 1 h at 37 °C before washing the cells with Phosphate Buffered Saline (pH 7.2) and transfecting each well with: 1 μg of pN, 1 μg of pP, 1 μg of pL and 2 μg of genome plasmid using FuGENE6 (Promega). Plates were incubated for 48–72 h at 37 °C prior to assessing if virus rescue had occurred by fixation and staining fixed cells with FITC conjugated anti-N antibodies (Fujirebio, Malvern, PA, USA). Where successful rescue events were observed, virus was passaged on BHKs until 100% infectivity was reached and titrated as previously described [20]. Virus titres were determined as focus forming units per mL (ffu/mL). Multistep growth curves were conducted as previously described [21]. To generate growth curves, 100 µL of supernatant was harvested and frozen at −80 °C for each time point required. Viruses were thawed and titrated in triplicate on BHK cells to determine titres at each time point.

### 2.3. In Vivo Experimentation

All in vivo experimentation was carried out within ACDP3/SAPO4 biocontainment facilities at the Animal and Plant Health Agency (APHA), Weybridge, UK. Mouse experimentation was undertaken in accordance with strict Home Office regulations under the Animals in Scientific Procedures Act (1986) under HO project license PPL70/7394. All experimentation was reviewed internally by the APHA Animal Welfare in Experimental Research Board (AWERB) prior to initiation. All naïve animals were sourced from licensed and registered breeder within the UK. All animals were given access to food and water ad libitum throughout all experimentation and animals were checked at least twice daily to assess welfare and the development of clinical disease.

Three to four week old CD1 mice were purchased (Charles River, Wilmington, MA, USA) and microchipped using Trovan chips to enable identification. Mice were vaccinated using the human rabies vaccine VeroRAB (Novartis, Basel, Switzerland) at a 1 in 20 in sterile filtered deionised water following reconstitution as per the manufacturer’s instructions. Mice were vaccinated via the intraperitoneal route with 0.5 mL of diluted vaccine to the lower right hand quadrant of the abdomen on days 0 and 14. Mock vaccinated animals were included for each challenge group to ensure the validity of the challenge virus. At 21 days post vaccination, the dorsal tail vein of each mouse was nicked under anaesthesia using a scalpel blade and blood was collected in CB300 tubes (Sarstedt, Hampton, NH, USA). Following collection, blood samples were stored at 4 °C overnight prior to centrifugation at 2500 rpm (860× *g*) for 10 min and serum separated from the blood pellet. Serum samples were heat inactivated at 56 °C for 30 min and stored at −20 °C until required.

Naïve mice were challenged intracranially (IC) with 100 ffu/30 µL of infectious recombinant virus. Following infection, mice were checked twice daily and clinical scores were recorded for each mouse according to an established clinical score system with defined humane end points [26]. For assessment of peripheral pathogenicity, mice were inoculated into the left hind footpad (FP) with 1000 ffu/50 μL. Mice were monitored for 28 days post infection. Results are only shown for 12 days for clarity as no further animals succumbed during the remainder of the experiment. Clinical signs were scored and mice terminated as described [26]. The humane endpoint for termination and cervical dislocation was a clinical score of 3. Mice were cardiac bled under terminal anaesthesia prior to termination.

### 2.4. Serology

At 21 days post vaccination, mice were tail bled for assessment of seroconversion to vaccine. Each serum sample was run on a partial dilution series using a rabies pseudotype neutralisation assay (PNA) as described previously [27] ranging from a 1 in 20 to 1 in 640 dilution, due to the limited volume of serum. This enabled determination of whether the mice had seroconverted to a titre comparable to the WHO standard 0.5 IU/mL serum. Following the duration of the challenge experimentation mice were cardiac bled under terminal anaesthesia and sera assessed by fluorescent antibody virus neutralization (FAVN) and modified (m) FAVN as described previously [11,18].

## 3. Results

**Figure 1:**
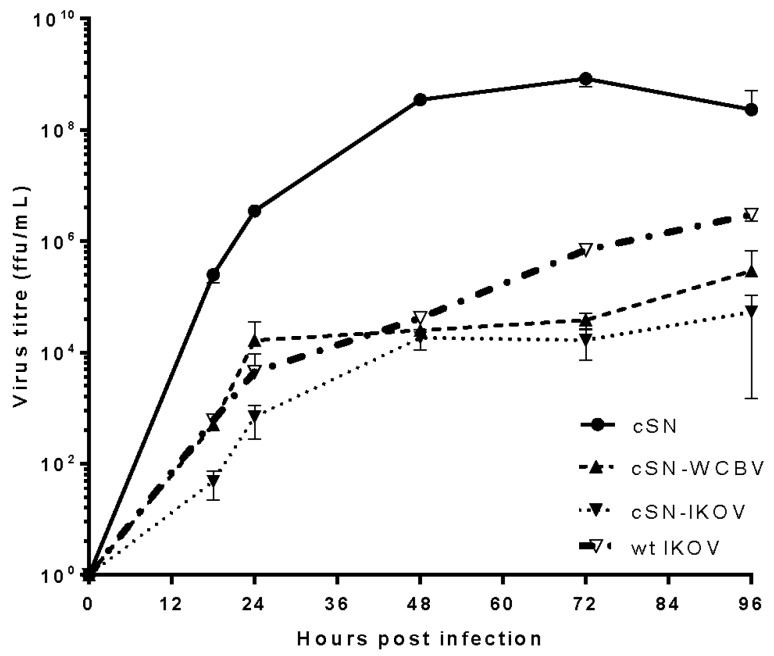
Growth kinetics of recombinant lyssaviruses in vitro. Multiple step growth curves of each virus starting from MOI 0.01.

Following rescue, cSN-IKOV reached 100% infection at passage 6 and cSN-WCBV by passage 7. The final passage of the rescued virus was compared with the original clone and no mutations were detected. The peak titres of the recombinant viruses reached titres comparable to previously generated recombinants that used the same vaccine backbone with the heterologous G from the European bat lyssaviruses (EBLVs) [21]. Growth curves showed a reduction in growth kinetics compared but recombinant viruses were viable and grew successfully (Figure 1). The wildtype WCBV was unavailable for comparison and so the chimeras were compared against the parent cSN vaccine and the IKOV wildtype virus. There was a notable difference between the growth curve of wildtype cSN and the recombinant viruses (Figure 1). The wildtype vaccine cSN strain grew to a peak titre of 8.35 × 10^8^ ffu/mL at 72 h post infection (hpi) whilst wildtype IKOV grew to 3.6 × 10^6^ ffu/mL after 96 h. The peak titre of the next most successful virus; cSN-WCBV-G was 2.92 × 10^5^ ffu/mL at 96 hpi. All viruses were detectable by 18 hpi. The end point titre of cSN-IKOV-G was 5.33 × 10^4^ ffu/mL, 2 logs lower than the wildtype IKOV. The growth patterns of each virus also differed. The growth of cSN had plateaued by 96 hpi. In contrast cSN-WCBV-G and cSN-IKOV-G both appeared to be increasing in titre over time as both peaked at 96 hpi. This may indicate a retarded growth rate of these two recombinants.

The degree of protection afforded by a human rabies vaccine to the recombinant viruses was then assessed in vivo. Blood collected from vaccinated mice were used in a pseudotype neutralisation assay. The WHO control serum sample had a reciprocal titre of 639.99 on the PNA. The mock vaccinated pool 1 had a titre of 79.99 and the mock vaccinated pool 2 had a titre of just 11.31 indicating that none of these mock vaccinated mice had seroconverted. From the vaccinated mice, four mice seroconverted to the same titre as the WHO control; 639.99 with the remainder having even higher titres (>905.09).

**Figure 2:**
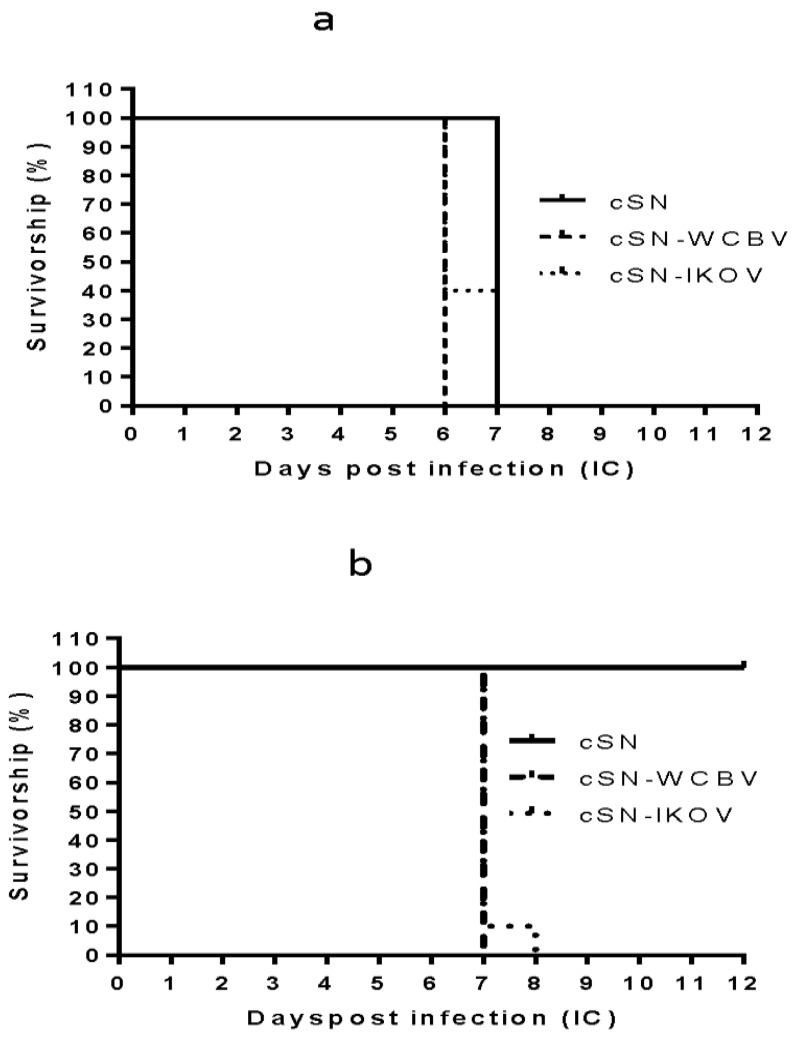
Survival curve of mice challenged IC. (**a**) The survival curve of mock vaccinated mice (*n* = 5 per virus). (**b**) The survival curve of mice vaccinated with VeroRAB and challenged with cSN, cSN-WCBV-G, or cSN-IKOV-G (*n* = 10/virus). Each mouse was challenged with 100 ffu/30 μL of virus via the intracranial route. Mice were observed for 28 days but results only shown up to day 12 for clarity as after this point no further mice developed clinical disease.

**Figure 3:**
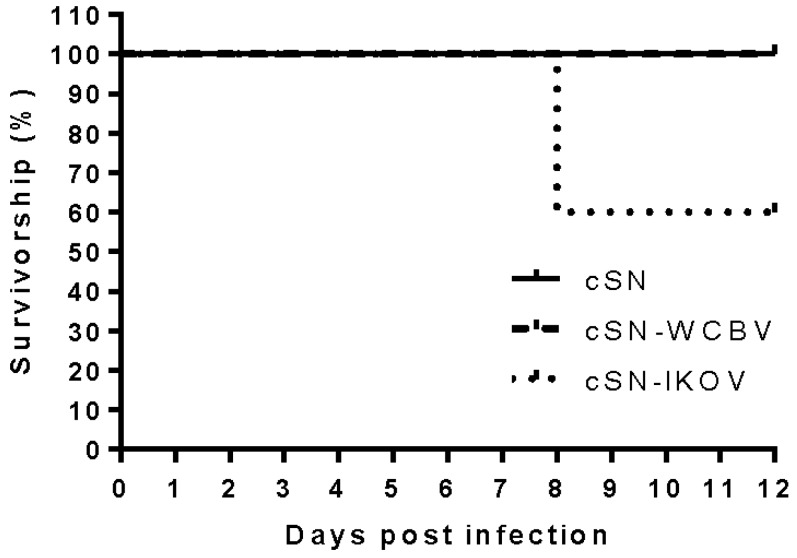
Survival curve of mice challenged peripherally. Each mouse was challenged with 1000 ffu/50 μL of either cSN, cSN-WCBV-G, or cSN-IKOV-G via the foot pad. (*n* = 5/virus). Mice were observed for 28 days. Results are shown up to day 12 after which no further mice developed clinical disease.

Two groups of 10 vaccinated mice and 5 mock vaccinated mice were challenged, at day 28 post vaccination, via the IC route with 100 ffu/mL of virus in 30 μL. Mice could not be challenged with wildtype WCBV as this isolate was not available to us. Infection with the wildtype IKOV had already been assessed and as such peripheral pathogenicity with this isolate was not assessed in this study [17]. All unvaccinated mice challenged IC with cSN, cSN-WCBV-G, or cSN-IKOV-G succumbed by day 6 or 7 (Figure 2a). From the groups of vaccinated mice, all those challenged IC with cSN survived challenge whilst those challenged with cSN-WCBV-G had to be terminated by day 7. In addition, 90% of the cSN-IKOV-G infected mice were terminated on day 7 and the remaining mouse was terminated with clinical disease that had reached the humane endpoint by day 8 (Figure 2b). To assess peripheral pathogenicity, each of the recombinant viruses was inoculated peripherally as described into naïve mice. With the exception of two cSN-IKOV-G inoculated mice that developed clinical disease and were humanely terminated on day 8, all mice survived to the end of the study (Figure 3).

## 4. Discussion

Novel lyssaviruses continue to be discovered globally. With the increasing recognition of the importance of bats as reservoirs of zoonotic pathogens, the popularity of leisure activities that facilitate human interactions with bats (e.g., caving, potholing, speleology), and the ongoing encroachment of humans into wild areas, the investigation of bat borne zoonotic pathogens is of relevance to human and animal health. For lyssaviruses, the zoonotic threat from bats is heightened as although not readily transmitted, where infection does occur and clinical disease develops, lyssavirus infection is invariably fatal. A further risk from these pathogens is through the potential for occupational exposure. Although lyssaviruses are principally spread through the bite of an infected animal, rare exposures including via the aerosol route and through exposed mucous membranes have been reported following laboratory or nosocomial infections [28,29]. In the laboratory, the potential for a needle stick injury exists and as such work with highly divergent lyssaviruses is limited to essential procedures. To this end, we used reverse genetics to evaluate recombinant viruses containing divergent glycoproteins as surrogates for wildtype viruses as an alternative to using wildtype lyssaviruses of undefined human pathogenicity in vaccination challenge experimentation. Construction and rescue of recombinant viruses based on the cSN (SAD-B19) vaccine strain of rabies enabled the generation of viruses containing glycoproteins of WCBV and IKOV. Existing rabies vaccines are not generally considered able to induce neutralising antibodies that can effectively protect against these viruses and as such the attempted development of a potentially bio-safe alternative is warranted. This approach has further demonstrated that where live virus isolates are not available, as was the situation with WCBV in this study, gene synthesis can be used to make a recombinant virus expressing the glycoprotein. The study also demonstrated that homologous M and G are seemingly not necessary for successful virus rescue even where interactions between highly divergent proteins are required. Certainly, swapping the G alone from divergent viruses into a vaccine strain backbone demonstrated that homologous M and G proteins were not required for virus rescue. The most recently described Lleida Bat Lyssavirus (LLEBV) was not investigated as part of this study as the sequence of the G was not available although more recently it has been defined [30] and as such further work may include assessment of LLEBV G in a similar system [16]. From a neutralisation perspective, these chimeric viruses have great utility in assessment of in vitro serological neutralisation although sera specific for each virus is necessary to thoroughly investigate the cross reactivity of antibody responses to different lyssavirus species. Other studies have suggested a utility in pseudotype viruses although neutralisation profiles are often inflated using this approach as the surrogates do not necessarily reflect outcomes with live viruses [31].

Initially, virus rescue and in vitro replication was assessed. Whilst the growth of the parent cSN vaccine strain was clearly greater than that of the two chimeras generated, their peak titres were similar to that observed for other lyssaviruses where the generation of high titre virus stocks is considered to be problematic [21]. The peak titre achieved for cSN-IKOV was lower than observed for wildtype IKOV suggesting that the recombinants are likely attenuated compared to wildtype viruses as expected. Where elements of divergent lyssaviruses are swapped into heterologous backbones the effect of having heterologous M and G proteins is of interest. Certainly, it has been shown for a number of *Mononegavirales* including respiratory syncytial virus, influenza, and vesicular stomatitis virus that M interacts directly with G to enable high efficiency viral budding from host cells [32,33,34]. For rabies, the interaction between G and M enables efficient budding of viral particles [35] with the interacting domain residing in the cytoplasmic domain of G [32,36]. Indeed, the lyssavirus M protein regulates the viral life cycle, inhibiting transcription and even stimulating replication when supplied in trans [37]. Further, the replacement of non-pathogenic M proteins with those from pathogenic strains has resulted in an increase in virulence [38]. This effect was also demonstrated whereby a significant increase in pathogenicity was demonstrated when including homologous M and G [24]. Critically, studies have demonstrated that the exchange of G ectodomains alone between lyssaviruses enables virus rescue [36]. In contrast, in the present study, rescue is tolerated where complete G ORFs are exchanged with little effect on replicative ability in vitro even where exchanges are made between the most divergent lyssaviruses. Different studies have demonstrated that other lyssavirus G proteins can be swapped into the cSN backbone with similar outputs [21] although these studies assessed lyssaviruses more closely related to RABV with a greater degree of sequence identity in G. The process by which protein structures affect these interactions cannot be concluded although the conservation seen across the lyssavirus M protein at least suggests that this group of viruses have evolved with similar drivers for genetic conservation.

From the in vivo studies, data from the survival curves indicated some substantial differences between survivorship in intracranially challenged vaccinated and unvaccinated individuals. It has been previously reported that cSN is pathogenic when inoculated via the IC route but is considered to be apathogenic following peripheral inoculation [21]. All unvaccinated mice challenged IC with cSN were terminated with clinical disease by 7 dpi whereas all vaccinated mice challenged with these viruses survived as expected [11]. The development of clinical disease in mice infected with cSN-WCBV-G and cSN-IKOV-G, either those previously vaccinated or mock vaccinated, confirms the pathogenic role of the G proteins and supports existing data describing a lack of protection against divergent lyssaviruses from vaccine induced immunity [10]. Both vaccinated and unvaccinated mice challenged IC with cSN-WCBV-G developed clinical disease, being humanely terminated at 7 dpi. The same outcome was observed with the cSN-IKOV-G IC infected mice with all unvaccinated mice developing clinical disease and being terminated by day 6 post infection. Further, 90% of the vaccinated mice were terminated with clinical disease by 7 dpi with the remaining 10% developing clinical disease that required termination by 8 dpi. This confirmed the lack of protection afforded by rabies vaccines against these divergent lyssaviruses and mimics in vitro data suggesting that strong neutralising antibodies induced by the rabies vaccines are unable to neutralise these viruses.

The peripheral infection of naïve mice with cSN, cSN-WCBV-G, and cSN-IKOV-G demonstrated a lack of pathogenicity of all viruses following peripheral inoculation with the exception of 40% (*n* = 2/5) of the cSN-IKOV-G mice that developed clinical disease by day 8 and had to be humanely terminated suggesting that a degree of pathogenicity is conferred by the IKOV G protein. Few studies assessing pathogenicity of WCBV and IKOV have been reported. For WCBV, inoculation of the big brown bat (*Eptesicus fuscus*) demonstrated that infection via the oral route was non-viable with no clinical or serological response being detected and peripheral inoculation into the masseter muscle causing only a serological response. In contrast, clinical disease developed in bats inoculated with virus in the musculature of the neck with 57% succumbing to infection and the remainder generating a serological response demonstrating exposure [39].

For IKOV, murine studies have shown that both peripheral and IC inoculation of wildtype IKOV causes clinical disease. Interestingly, whilst neat (10^4.8^TCID_50_/mL) IC inoculation and peripheral inoculation caused 100% mortality, a detectable dose effect was reported following intramuscular infection with a 10-fold dilution of IKOV with only 40% of mice infected peripherally succumbing to infection [17]. The lower dose used in this study equates to an inoculation of approximately 190 infectious particles. In comparison, in this study infection with a higher dose of 1000 ffu/50 µL led to fewer animals developing clinical disease and as such it is reasonable to conclude that the vaccine expressing the IKOV G may be less pathogenic than the wildtype virus, especially when considering the growth curve data whereby the recombinant virus growth kinetics were retarded when compared with the cSN virus and the IKOV wildtype isolate. Regardless, the effect of route of inoculation requires further assessment and ideally an assessment of peripheral pathogenicity with divergent wildtype viruses is warranted. A minimal dose for infection with lyssaviruses has not been defined although it has to be assumed that if a single virion enters a nerve and establishes infection then this is sufficient to cause disease. Certainly, effects of dose have been assessed, and titrating out virus can lead to a reduction in pathogenicity. Previous murine and chiropteran studies that titrated out lyssaviruses to assess the outcomes of multiple exposures to low doses of virus demonstrated the effect of dose [40,41]. However, in contrast, other bat models of infection have demonstrated less survivorship where low doses of virus are inoculated for some lyssaviruses [42,43,44]. Some standardisation of challenge models would enable better comparison between experiments with these viruses.

As described, the use of recombinant chimeric viruses as a substitute for wildtype lyssaviruses for in vivo vaccination challenge experimentation may represent an alternative option to wildtype viruses that may even reduce operator risk from these lethal viruses. The opportunity to utilise such constructs as vehicles for vaccine development may also be of future use as it is likely that the expression of the divergent glycoprotein is sufficient to generate a neutralising immune response that may be protective against the chosen glycoprotein. Certainly, whilst the neutralising antibody response to vaccination is understood to be the key to protective immunity, the cell mediated responses to elements of the viral particle other than the glycoprotein requires further interrogation, and such recombinants may aide that. Further assessment of such constructs, including mutagenesis of residues within G that are associated with viral pathogenesis, could also extend these studies [20,45,46,47,48]. Finally, the observation that gene swaps between highly divergent lyssaviruses are tolerated may influence the future direction of research assessing these high-risk pathogens for which no vaccine protection or post exposure tools are available.

## Supplementary Materials

The following are available online at http://www.mdpi.com/1999-4915/10/3/130/s1, Figure S1: Schematic of full length plasmids constructed containing heterologous glycoproteins. The recombinant DNA clones of each genome are shown with the glycoprotein being swapped to contain divergent G proteins in place of the wildtype protein. Restriction enzymes utilised to swap each gene are labelled.
