# Supplementary figures and images for: Utilisation of Chimeric Lyssaviruses to Assess Vaccine Protection against Highly Divergent Lyssaviruses
Source: Viruses. 2018 Mar 15;10(3):130. doi: 10.3390/v10030130 (PMC5869523; doi:10.3390/v10030130)

Evans et al., 2017 Supplementary figure 1

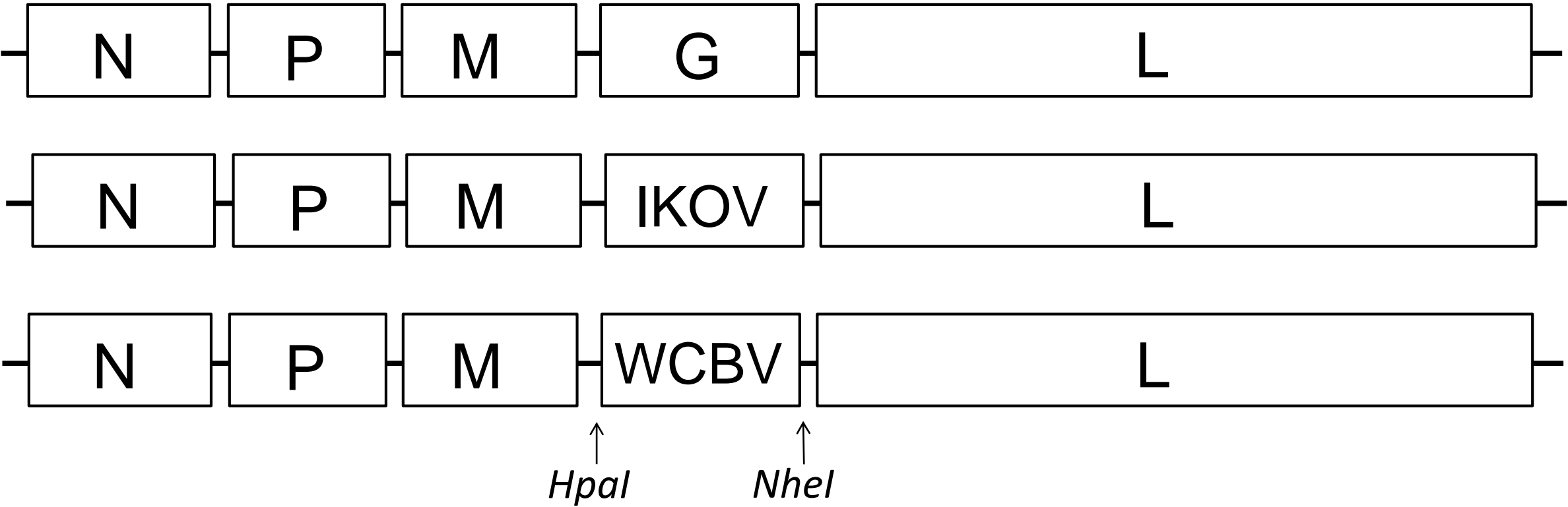

Evans et al., 2017- Supplementary file 2

a) IKOV

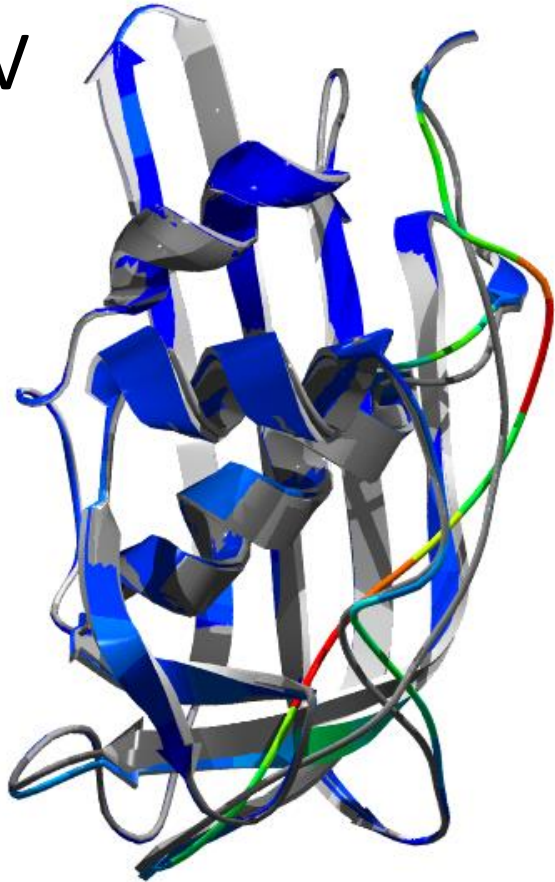

b) WCBV

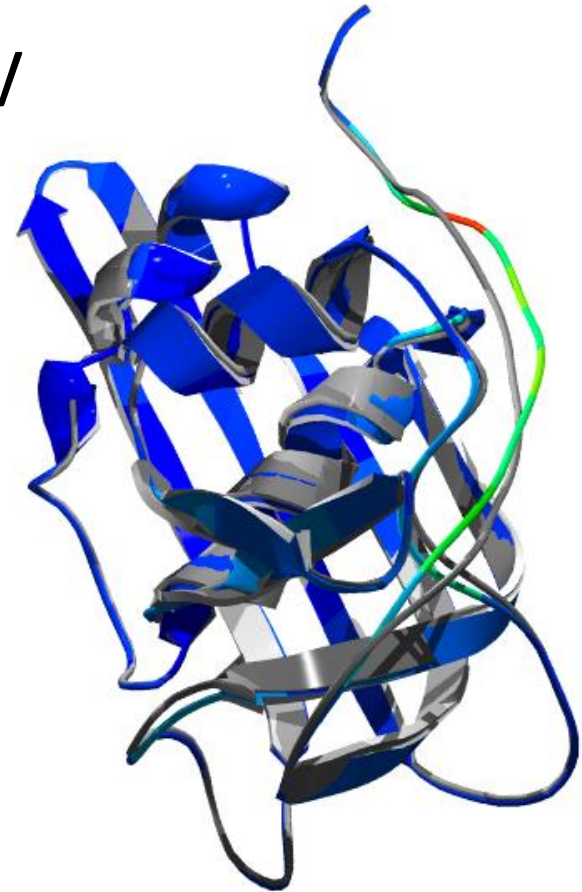

c) LLEBV

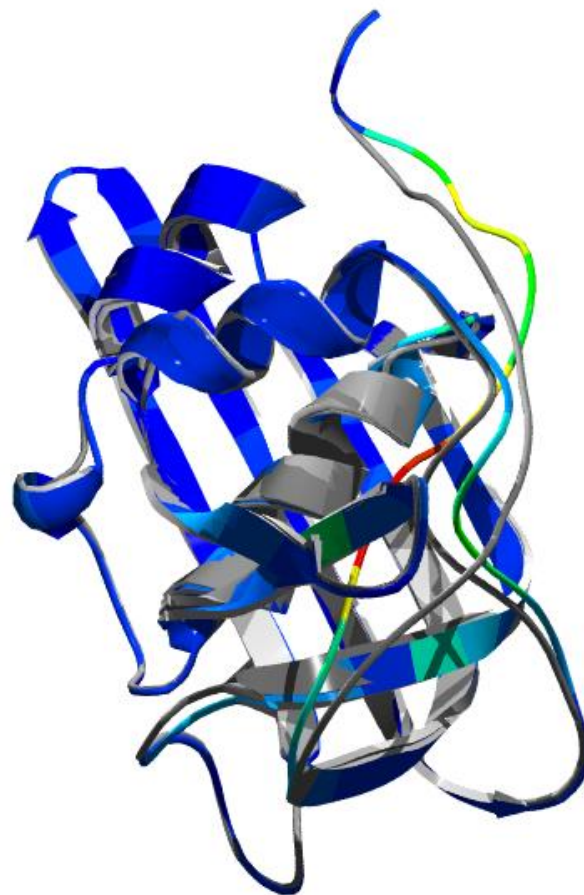

Supplement: Supplementary file 1 [file viruses-10-00130-s001.pdf]
